# Inhibition of Starvation-Triggered Endoplasmic Reticulum Stress, Autophagy, and Apoptosis in ARPE-19 Cells by Taurine through Modulating the Expression of Calpain-1 and Calpain-2

**DOI:** 10.3390/ijms18102146

**Published:** 2017-10-14

**Authors:** Yuanyuan Zhang, Shu Ren, Yuci Liu, Kun Gao, Zheng Liu, Zhou Zhang

**Affiliations:** Department of Pharmacology, Shenyang Pharmaceutical University, Shenyang 110016, China; yuanyuanzhangyyz@163.com (Y.Z.); shuyeah829@163.com (S.R.); yuciiliu@163.com (Y.L.); Gaokun@sinqi.com (K.G.)

**Keywords:** ARPE-19, autophagy, ER stress, apoptosis, taurine, calpain-1, calpain-2, age-related macular degeneration

## Abstract

Age-related macular degeneration (AMD) is a complex disease with multiple initiators and pathways that converge on death for retinal pigment epithelial (RPE) cells. In this study, effects of taurine on calpains, autophagy, endoplasmic reticulum (ER) stress, and apoptosis in ARPE-19 cells (a human RPE cell line) were investigated. We first confirmed that autophagy, ER stress and apoptosis in ARPE-19 cells were induced by Earle’s balanced salt solution (EBSS) through starvation to induce RPE metabolic stress. Secondly, inhibition of ER stress by 4-phenyl butyric acid (4-PBA) alleviated autophagy and apoptosis, and suppression of autophagy by 3-methyl adenine (3-MA) reduced the cell apoptosis, but the ER stress was minimally affected. Thirdly, the apoptosis, ER stress and autophagy were inhibited by gene silencing of *calpain-2* and overexpression of *calpain-1*, respectively. Finally, taurine suppressed both the changes of the important upstream regulators (calpain-1 and calpain-2) and the activation of ER stress, autophagy and apoptosis, and taurine had protective effects on the survival of ARPE-19 cells. Collectively, this data indicate that taurine inhibits starvation-triggered endoplasmic reticulum stress, autophagy, and apoptosis in ARPE-19 cells by modulating the expression of calpain-1 and calpain-2.

## 1. Introduction

Age-related macular degeneration (AMD) is a progressive retinal degenerative disease affecting more than 150 million people in the world [1,2]. Disruption of retinal pigment epithelium (RPE) is one of the major pathological changes in AMD. There are some factors believed to be involved in the development of RPE damage, such as apoptosis, mitochondrial dysfunction, lack of nutrients and oxygen, and calcium overload [3,4,5]. However, the exact mechanism is still unknown. Cells with nutrient deficiencies have been found to be hypersensitive to various cell death stimuli, such as autophagy [6], loss of calcium homeostasis [7], and ER stress [8,9]. Autophagy is the major intracellular degradation system that regulates the degradation of long-lived proteins, organelles, and other cellular contents [10]. This process occurs at basal levels in all cells but it is rapidly upregulated in response to nutrient deprivation, endoplasmic reticulum (ER) stress, pathogen infection, and/or hypoxia [11]. ER stress is known to cause autophagy [12,13]. Autophagy activation leads to the reduction of accumulation of misfolded and aggregated proteins in several neurodegenerative disorders. Conversely, the over activation of autophagy can trigger neuronal cell death following ethambutol injury in rat retina [14]. Autophagy-mediated apoptosis has attracted much attention [15,16,17].

ER is a major organelle responsible for the translation, folding, and transport of proteins for local cellular use, intracellular calcium homeostasis, as well as cell death [18]. Physiological and pathological conditions that disrupt protein folding and/or cause loss of calcium homeostasis leading to ER dysfunction include ischemic injury, nutrition deprivation, and diabetic retinopathy [19,20,21]. Cells react to ER stress by activation of the unfolded protein response (UPR) pathway, which is a cellular protective response, but the use of excessive or prolonged UPR can kill cells by induction of apoptosis. UPR results in the induction of molecular chaperone GRP78 and the ER-resident caspase-12, Chop, whose activation has been proposed to be mediated by calpain and caspase processing, although their relative contribution remains unclear. However, if ER homeostasis is not restored in a timely manner its functional capacity is rapidly exceeded and results in apoptosis [8,22].

Calpains are calcium-dependent proteases that perform important roles in both physiological and pathological processes in neurodegenerative disease [7,23]. The main calpain isoforms in the brain are μ-calpain (calpain-1) and m-calpain (calpain-2) [24]. Some studies have indicated that, under certain conditions, calpain activation could cause tissue damage and cell death [25,26]. However, some studies showed that calpain-1 and calpain-2 play opposite roles in retinal ischemia/reperfusion injury but both, calpain-1 and calpain-2, provide neuroprotection in chronic myeloid leukemia [27,28]. Furthermore, recent analysis has established a role for calpain-mediated cleavage of beclin-1 and the deregulation of basal autophagy in rat retina following ischemia/reperfusion injury [23]. Previous studies have also shown that calpain-2 activation is not only regulated on the ER membrane but also plays a role in ER stress-induced apoptosis and β cell death [29]. Based on previous studies, calpains are the important upstream regulators of autophagy and ER stress.

Taurine is one of the most abundant amino acids in ocular tissues [30], such as the cornea, iris, lens, and ciliary bodies. Taurine can carry out multiple functions in cells: ER stress response, autophagy modulation, anti-apoptosis, and so on [31,32,33]. Another important function of taurine is the modulation of calcium influx, which may contribute to its neuroprotective effects [34]. However, recent studies have not demonstrated the relationship between taurine and Earle's balanced salt solution (EBSS)-induced cell injuries.

**Figure 1:**
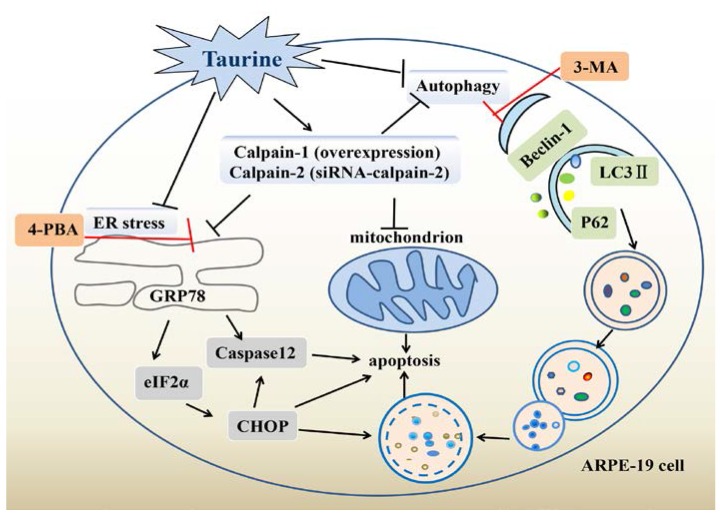
Schematic diagram of the protective effects of taurine in ARPE-19 cells. In starved cells, the calcium homeostasis was eliminated, and autophagy, endoplasmic reticulum (ER) stress and apoptosis were activated. Furthermore, taurine inhibits starvation-triggered endoplasmic reticulum stress, autophagy, and apoptosis in ARPE-19 cells by modulating the expression of calpain-1 and calpain-2. The red & black T-bars stands for inhibition.

In this study, we showed that EBSS-triggered starvation activates ER stress, autophagy and apoptosis in cells. We also explored the role of calpains in EBSS-induced cell injuries. Furthermore, taurine inhibits starvation-triggered endoplasmic reticulum stress, autophagy, and apoptosis in ARPE-19 cells by modulating the expression of calpain-1 and calpain-2 (Figure 1).

## 2. Results

### 2.1. Effects of EBSS Treatment for Different Durations on Expression of Calpains and Autophagy-Relative Proteins in ARPE-19 Cells

**Figure 2:**
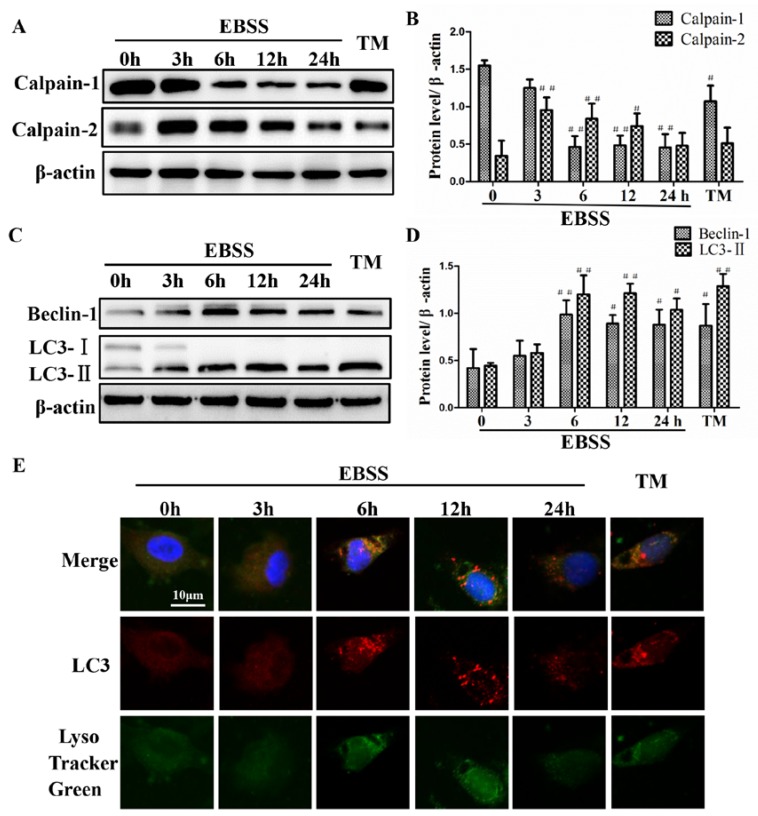
Earle’s balanced salt solution (EBSS) increases calpain activity and autophagy in ARPE-19 cells in a time-dependent manner. (**A**,**B**) Western blot analysis was performed to detect the expression levels of calpain-1 and calpain-2 in ARPE-19 cells. (**C**,**D**) Autophagy was evaluated by detecting LC3-II expression with immunoblots. (**E**) The colocalization of LC3 and LysoTracker Green in ARPE-19 cells. Scale bar = 10 μm, TM stands for tunicamycin. # *p* < 0.05, ## *p* < 0.01 compared with control (0 h).

To assess whether the EBSS treatment influences the activities of calpains in ARPE-19 cells, the expression of calpain-1 and calpain-2 were detected by Western blot. The results demonstrate that after EBSS treatment, the protein expression of calpain-1 is decreased from 3 h until 24 h, and calpain-2 is increased over the first 3 h and then gradually decreased (Figure 2A,B). Autophagy was evaluated by detecting LC3-II and beclin-1 expression. The protein expression levels of beclin-1 and LC3-II were gradually increased over the 3 h followed by a dramatic decrease from 6 h until 24 h (Figure 2C,D). The activity of LC3-II within the autolysosome has been proposed as one of the autophagic flux assays. Moreover, the dynamics of LC3-II and lysosomal acidity were markedly increased after a 6 h treatment with EBSS (Figure 2E). Tunicamycin treated ARPE-19 cells served as positive control. Our results indicate that starvation induces the activities of calpains and autophagy. Additionally, these changes could be fully activated by 6 h and subsequently this time point was selected for our experiments.

### 2.2. Effects of EBSS Treatment for Different Durations on Expression of ER Stress-Relative and Apoptosis-Relative Proteins in ARPE-19 Cells

**Figure 3:**
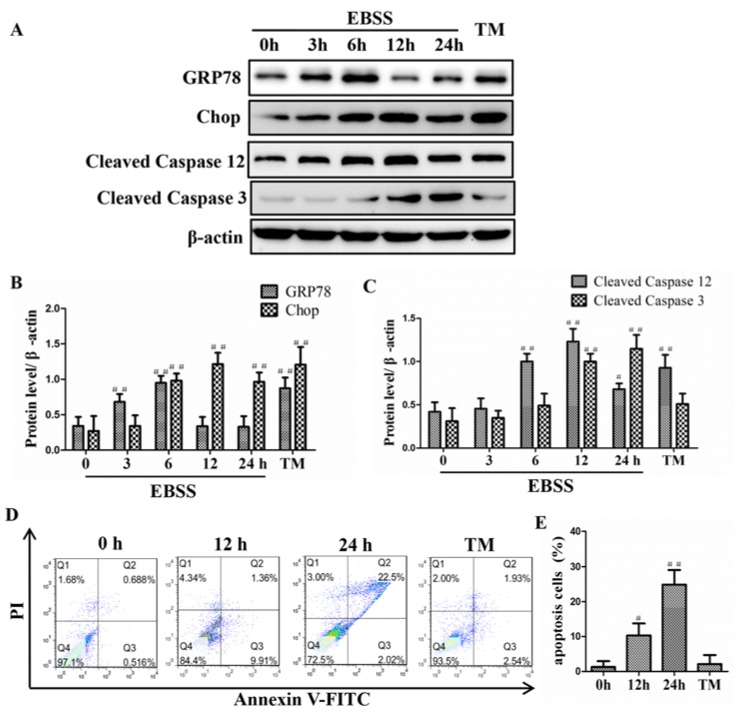
EBSS induced ER stress and apoptosis in ARPE-19 cells in a time-dependent manner. (**A**–**C**) The ER stress-related proteins and the ER stress-associated downstream apoptotic protein caspase-3 (a mitochondrial apoptotic marker) were examined by Western blot; (**D**,**E**) The percentage of apoptotic cells was determined based on a flow cytometric analysis. The data are presented as the means ± SD of three independent experiments. TM stands for tunicamycin. # *p* < 0.05, ## *p* < 0.01 compared with control (0 h).

Autophagy is known to be induced by ER stress. To test if EBSS-induced autophagy involves an ER response, we examined cell lysate from ARPE-19 cells for ER chaperone proteins GRP78, Chop, and caspase-12 by immunoblot analysis. When compared to controls, the expression levels of GRP78, Chop, and cleaved caspase-12 were significantly elevated by 3 h until 6 or 24 h after EBSS treatment. Moreover, a mitochondrial apoptotic marker caspase-3 was also activated by 12 h and the level of caspase-3 was sustained until 24 h (Figure 3A–C). The cell apoptosis rate was markedly increased at 24 h (Figure 3D,E). Tunicamycin group was used as positive control for these experiments.

Our results indicated that the ER stress and apoptosis could be induced at 6 and 12 h, respectively. Therefore the two timepoints were selected for our subsequent experiments. Furthermore, the activation of endoplasmic reticulum stress precedes apoptosis.

### 2.3. Inhibiting ER Stress and Autophagy Attenuated EBSS-Induced Apoptosis in ARPE-19 Cells

**Figure 4:**
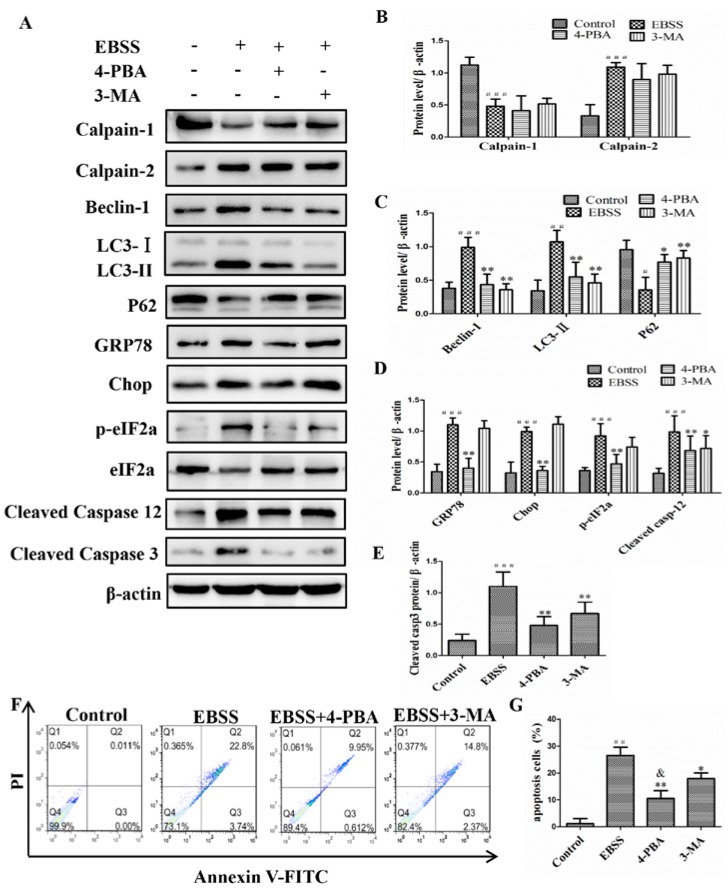
Inhibition of ER stress and autophagy attenuated EBSS-induced apoptosis in ARPE-19 cells. (**A**–**E**) The expression levels of calpain-1, calpain-2, ER stress-related proteins, autophagy-related proteins and the mitochondrial apoptotic marker caspase-3 were examined by Western blot. (**F**,**G**) The apoptosis rate of ARPE-19 cells was evaluated by a flow cytometer. # *p* < 0.05, ## *p* < 0.01, ### *p* < 0.001 compared with control. * *p* < 0.05, ** *p* < 0.01, compared with EBSS group, ^&^ *p* < 0.01 compared with 3-MA group.

To further investigate the regulation relationships of ER stress, autophagy, and apoptosis, ARPE-19 cells were pretreated in the absence or presence of 4-PBA (1 mM) or 3-MA (1 mM) inhibitors for 1 h and then treated with EBSS for 6 h. We found that EBSS caused a significant increase in the expression of calpain-2, beclin-1, LC3, GRP78, p-eIF2α, Chop, caspase-12, and caspase-3 (treated with EBSS for 12 h) (Figure 2 and Figure 3). However, except for calpain-2, the levels of other proteins were noticeably decreased after 4-PBA pretreatment (Figure 4A–E), indicating that the ER stress pathway was involved in the activation of autophagy and apoptosis. To assess the potential involvement of the autophagy in ARPE-19 cells, the 3-MA inhibitor was used. The results show that pretreatment with 3-MA reverses the up-regulation of beclin-1 and LC3-II in response to EBSS. However, EBSS-induced activation of ER-related proteins was not influenced by 3-MA. The expression of calpain-1 was decreased in EBSS treatment and had a slight decrease after being pretreated with 4-PBA (1 mM) or 3-MA (1 mM) inhibitor for 1 h. Our findings indicate that the activation of ER stress could promote autophagy and apoptosis. Furthermore, calpain may be a key upstream regulator of ER stress, autophagy, and apoptosis. Annexin V/PI double staining indicated that blockade of ER stress induction by 4-PBA or inhibition of autophagy by 3-MA resulted in a protection of ARPE-19 cells. In addition, ER stress plays a more important role than autophagy in cell apoptosis (Figure 4F,G). Our results show that autophagy may regulate apoptosis but not ER stress, and that autophagy and apoptosis together may be regulated by ER stress. Furthermore, ER stress plays a more important role than autophagy in cell apoptosis. In addition, the results illustrated in Figure 4 indicate that calpains may be key upstream regulators of ER stress, autophagy, and apoptosis.

### 2.4. Calpain-1 and Calpain-2 Played Key Roles in EBSS-Induced Cell Injuries

**Figure 5:**
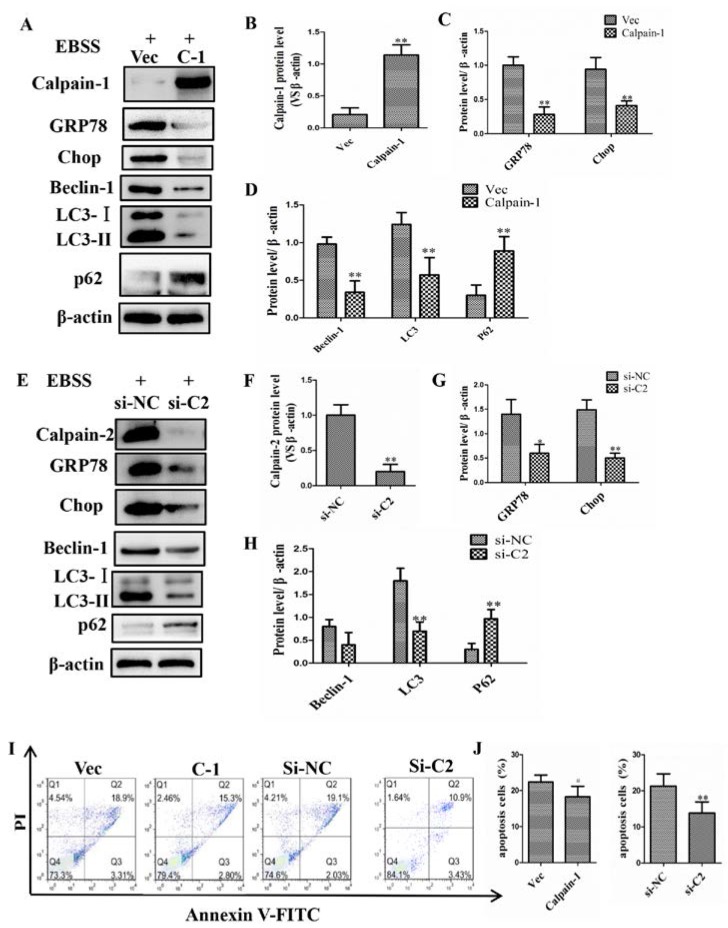
Calpain-1 and calpain-2 modulate ER stress, autophagy and apoptosis in ARPE-19 cells. (**A**–**D**) The activations of ER stress, autophagy and apoptosis were inhibited by *calpain-1* plasmid transfection; (**E**–**H**) The activations of ER stress, autophagy and apoptosis were suppressed by siRNA knockdown of *calpain-2*. (**I**,**J**) The apoptosis rate of ARPE-19 cells was evaluated by a flow cytometer. * *p* < 0.05, ** *p* < 0.01, compared with EBSS group, (C1 = calpain-1, C2 = calpain-2).

To further examine whether EBSS-induced ER stress, autophagy and apoptosis are dependent on calpains, we used plasmid vector carrying *calpain-1* leading to a marked increase of calpain-1 protein level. When the cells were treated with *calpain-1* plasmid, EBSS treatment did not reduce calpain-1 activity, compared with EBSS groups in ARPE-19 cells. To further validate whether calpain-1 is involved in EBSS-triggered ER stress, autophagy and apoptosis, we examined the protein levels of GRP78, Chop, caspase-12, Beclin-1, LC3, P62, and caspase-3 when calpain-1 activity was improved with *calpain-1* plasmid. The results show that ER stress, autophagy and apoptosis were inhibited by the overexpression of *calpain-1* (Figure 5A–D). Meanwhile, siRNA knockdown of *calpain-2* had a similar effect on the regulation of ER stress, autophagy, and apoptosis (Figure 5E–H). Our data indicate that the activation of ER stress, autophagy, and apoptosis were mainly due to the activation of calpain-2 and the suppression of calpain-1. We further examined the efficacy of the overexpression of *calpain-1* and the siRNA knockdown of *calpain-2* on EBSS-induced apoptosis in ARPE-19 cells. We found that the apoptosis rate of ARPE-19 cells was reduced compared with that of the EBSS group. Moreover, siRNA knockdown of *calpain-2* might produce better protection than the overexpression of *calpain-1* (Figure 5I,J). These results suggest that calpains are the main upstream regulators of autophagy, ER stress, and apoptosis.

### 2.5. Effects of Taurine Treatment on Expression of Calpains and Autophagy-Relative Proteins Induced by EBSS in ARPE-19 Cells

**Figure 6:**
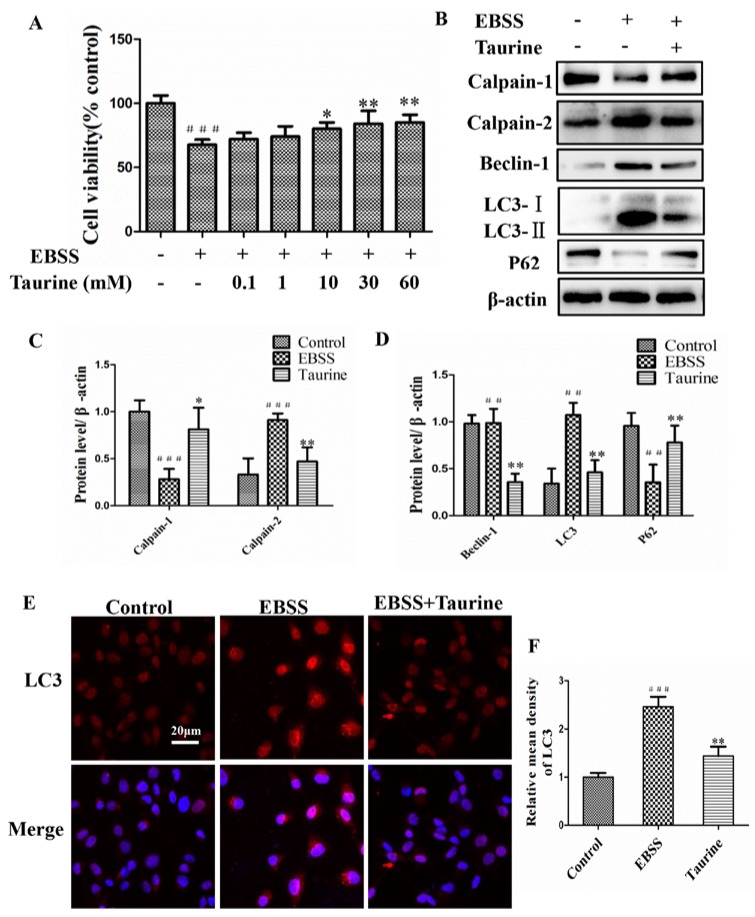
Effects of Taurine treatment on expression of calpains and autophagy-relative proteins induced by EBSS in ARPE-19 cells. (**A**) Cells were treated with 0.1–60 mmol/L taurine for 24 h. Cell viability was evaluated via CCK-8 assays; (**B**–**D**) Western blot analysis was performed to detect the expression levels of calpain-1, calpain-2 and autophagy-related proteins in ARPE-19 cells; (**E**,**F**) Confocal images of ARPE-19 cells labelled with anti-LC3 (red) and nuclear-stained with DAPI (blue). Scale bar = 20 μm. ## *p* < 0.01, ### *p* < 0.001 compared with control. * *p* < 0.05, ** *p* < 0.01, compared with EBSS group.

Next, we also examined the protective effect of taurine on EBSS-exposed ARPE-19 cells and the mechanisms for the underlying effects of taurine. The results demonstrate that 1–60 mmol/L of taurine has a protection on cell viability (Figure 6A). Western blot analysis shows that the expression of calpain-2, beclin-1, and LC3 in the EBSS group was upregulated compared with the control group, but downregulated in the taurine + EBSS group compared with that of just the EBSS group. The expression of calpain-1 and p62 was significantly increased by the intervention with taurine (Figure 6B–D). Autophagy was also evaluated by detecting LC3-II expression with immunofluorescence. The level of LC3-II was gradually increased in the EBSS group; taurine could reverse this phenomenon (Figure 6E,F). Our studies show that taurine blocked both changes in calpain levels and autophagy.

### 2.6. Effects of Taurine Treatment on the Expression of ER Stress-Relative and Apoptosis-Relative Proteins Induced by EBSS in ARPE-19 Cells

**Figure 7:**
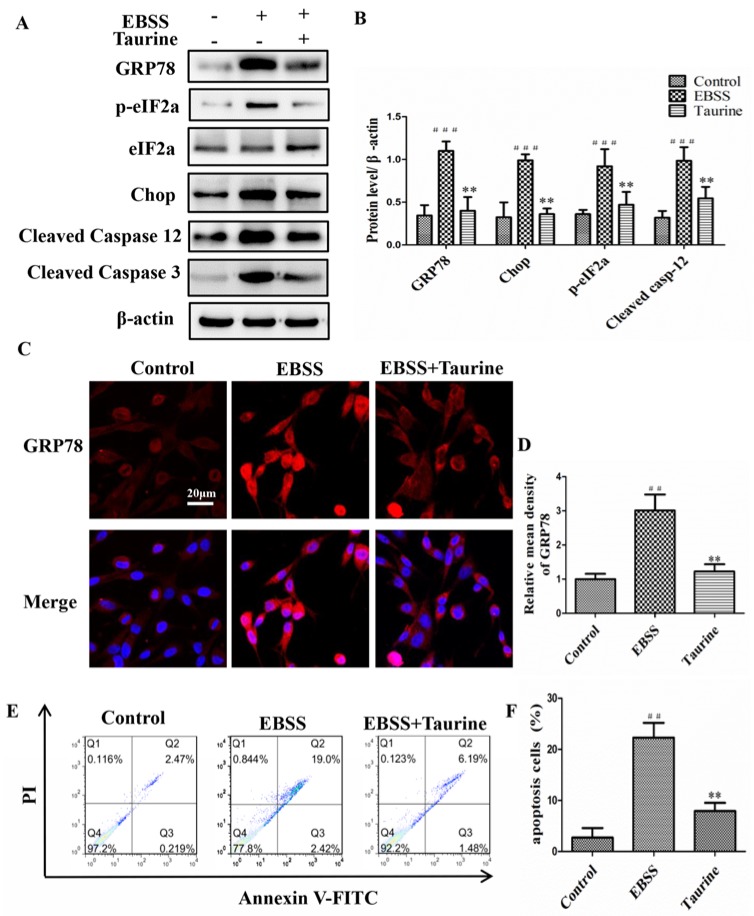
Effects of Taurine treatment on EBSS induced ER stress and apoptosis in ARPE-19 cells (**A**,**B**) The ER stress-related proteins and the ER stress-associated downstream apoptotic protein caspase-3 were examined by Western blot; (**C**,**D**) Confocal images of ARPE-19 cells labelled with anti-GRP78 (red) and nuclear-stained with DAPI (blue). Scale bar = 20 μm; (**E**,**F**) The percentage of apoptotic cells was determined based on a flow cytometric analysis. The data are presented as the means ± SD of three independent experiments. ## *p* < 0.01, ### *p* < 0.001 compared with control. ** *p* < 0.01, compared with EBSS group.

We also examined whether taurine affects ER stress and apoptosis. The expression of GRP78, p-eIF2α, Chop, cleaved caspase-12, and cleaved caspase-3 was down-regulated in the taurine+EBSS group when compared to controls treatment (Figure 7A,B). Moreover, ER stress was also evaluated by detecting GRP78 expressions with immunofluorescence. High fluorescence intensities in the EBSS group presented with aggregated fluorescent particles visible in the cytoplasm. Fluorescence intensity in the control and EBSS + taurine was relatively weak (Figure 7C,D). We found that taurine alleviated apoptosis induced by EBSS in ARPE-19 cells compared with the EBSS group (Figure 7E,F). These data together indicate that taurine suppressed both the changes in calpain levels and the activation of ER stress, autophagy, and apoptosis. Calpains are the important upstream regulators to autophagy, ER stress, and apoptosis. Therefore, taurine inhibits starvation-triggered endoplasmic reticulum stress, autophagy, and apoptosis in ARPE-19 cells by modulating the expression of calpain-1 and calpain-2 levels.

## 3. Discussion

In this study, we discovered that EBSS results in a number of effects on apoptosis including changes in calpain-1 and calpain-2 levels. Furthermore, we have demonstrated that the inhibition of starvation-triggered ER stress, autophagy, and apoptosis in ARPE-19 cells by taurine is caused by the modulation of the expression of calpain-1 and calpain-2 levels. Previous studies have shown that tunicamycin causes ER stress and autophagy in normal tissues and therefore we have selected it as positive control in our study [12,13].

Autophagy is essential for homeostasis of neuronal cells and its dysregulation is involved in several neurodegenerative disorders [35]. The level of conversion of LC3-I to LC3-II can be used as an indicator for autophagic activity [36,37]. The expression level of the autophagic marker LC3-II was higher in EBSS-induced ARPE-19 injuries. Our immunofluorescence results also indicate that LC3-II aggregated fluorescent particles are visible in the cytoplasm. Lysosome was also activated at the same time (Tracker Green) (Figure 2). Beclin-1 is the mammalian homolog of yeast Atg6 and is essential for recruitment of other Atg proteins during the early stages of autophagy [38]. The Western blot analysis revealed that the expression of beclin-1 after EBSS treatment was significantly higher than that of the control group in ARPE-19 cells (Figure 6). The p62 protein is selectively incorporated into autophagosomes through direct binding to LC3-II and is efficiently degraded in the autolysosome. Accordingly, the total p62 expression level is negatively correlated with autophagic flux [39,40,41]. The protein expression of p62 was much lower in EBSS groups compared to control groups (Figure 6).

Although autophagy can mediate both cell survival and death signaling, many studies in recent years focused on its role in the induction of apoptosis by various cellular stress factors. Indeed, we found that autophagy was important for cell apoptosis undergoing pharmacological ER stress. Blockade of ER stress induction by 4-PBA resulted in an influence on autophagy related proteins (Figure 4). Autophagy was also activated by calpains [6,23].

The glucose-regulated protein GRP78, a 78-kDa protein, also referred to as immunoglobulin heavy chain binding protein (BiP/GRP78), is a major molecular chaperone in the ER stress [42,43,44]. Phosphorylation of eiF2α is pivotal to control global rates of protein synthesis [12]. It has been reported that protein kinase R-like ER kinase (PERK) induces apoptosis via Chop accumulation under irremediable ER stress [22]. Caspase-12 is specifically involved in apoptosis, which results from the stress in the ER [45,46,47]. Our results show that ER stress signaling pathway (GRP78/Bip, eiF2α, Chop) is much higher in the EBSS groups compared to control groups indicating that EBSS induces ER stress. Specific inhibitors of ER stress signaling pathways IRE1, PERK, and ATF6 could be used to further investigate the main signaling pathway which is regulated by calpains and taurine.

The apoptosis marker caspase-3 was upregulated and the apoptosis rate was increased in the EBSS group (Figure 3), indicating that apoptosis occurs via mitochondria. Mitochondria are the main sites of biological oxidation in eukaryotic cells [48]. During cellular stress, Ca^2+^ was released from the ER into the cytoplasm. Ca^2+^ from the cytoplasm is taken up by mitochondria, and the accumulation of Ca^2+^ in mitochondria could stimulate the production of reactive oxygen species (ROS). ROS can further target ER-based calcium channels by triggering further release of Ca^2+^ leading to higher calcium load in the ER. This results in a vicious circle ultimately leading to apoptosis. The sustained high concentration of intracellular Ca^2+^, followed by activation of calpains, which critically mediates the subsequent ER-mitochondrial cross-talk [49,50,51,52,53]. Recent studies have shown that Ca^2+^ is associated with the formation of the autophagosome [54,55]. Therefore, activation of ER stress, the signaling pathway has been shown to correlate with mitochondria, autophagy, and calpains [29]. Moreover, the NOX2, NOX4, and NOX5 NADPH oxidases have been found localized in the ER where they are processed and activated [49,56], and NOX4 was found to mediate the UPR in response to ER stress, resulting in autophagy [57]. Interestingly, the NADPH oxidase (Nox) family proteins contributes to the production of ROS. ROS and calpain cascades can cross-talk with one cascade acting laterally on another cascade [49]. Therefore, the eliminated intracellular calcium homeostasis leading to apoptosis may involve autophagy and ER stress with redox enzyme activation. 

Most studies investigating the role of calpains in neurodegeneration have not addressed the respective roles of calpain-1 and calpain-2 and of the downstream cascades. Our results indicate that calpain-1 could inhibit the signaling pathways of ER stress and autophagy, while calpain-2 plays an opposite role in this study. These results are consistent with *calpain-1* overexpression or *calpain-2* knockdown (Figure 5). Calpains play important roles in increasing the cell survival by inhibiting ER stress and autophagy pathway.

Taurine is a sulfur free β-amino acid, in which the amino group is located on the β-carbon [34]. It is now widely accepted that taurine plays a crucial role in the development of many chronic diseases, such as age-related macular degeneration, diabetic retinopathy, and Alzheimer’s disease [58,59,60]. Taurine serves as a protective amino acid in the H9C2 cardiomyocyte, PC12 cells [31,33]. Our findings suggest remarkably protective effects of taurine on EBSS-induced ARPE-19 cells apoptosis by attenuating ER stress and autophagy via mechanisms involving the inhibition of calpain-2 and the activation of calpain-1 (Figure 6 and Figure 7).

ARPE-19 cells are widely used in the fields of apoptosis, autophagy and ER stress and the use of ARPE-19 cells allows us to get a basic understanding of protective mechanisms of taurine. The use of cell lines is not enough to reflect properties of the true physiological RPE. Therefore, primary RPE cells will be used in our future studies of the protective mechanisms of taurine. 

## 4. Materials and Methods

### 4.1. Cell Culture and Treatment

The human RPE cell line (ARPE-19, Shanghai GuanDao Biotech Co., Ltd., Shanghai, China) was cultivated in Dulbecco’s modified Eagle’s medium and F-12 nutrient mixture (Hyclone, Logan, UT, USA), supplemented with 10% FBS (Gibco, Grand Island, NY, USA), 100 U/mL penicillin, and 100 μg/mL streptomycin (Sigma-Aldrich, St.Louis, MO, USA). Cells were grown at 37 °C in a humidified atmosphere of 5% CO_2_. The medium was changed every 2 days. Cells that had grown to 80% confluence were used in the experiments. The cultures were exposed to media supplemented with EBSS for 0, 3, 6, 12, or 24 h or with tuicamycin (50 μM) as a positive control for 6 h. The cells were pretreated with 3-MA (1 mM) or 4-PBA (0.1 mM) for 1 h and then exposed to EBSS (6 or 12 h) for Western blotting or apoptosis detection (exposed to EBSS for 24 h).

### 4.2. Transfection Experiments

For overexpression experiments, ARPE-19 cells were transfected with a plasmid vector carrying *calpain-1* (1 μg) or an empty vector with lipofectamine 2000 reagent (Invitrogen, Carlsbad, CA, USA) according to the manufacturer’s protocol. For gene silencing of *calpain-2*, the specific siRNA against *calpain-2* was 5′-GAAGUGGAAACUCACCAAATT-3′; the control sequence was: 5′-UUCUCCGAACGUGUCACGUTT-3′. The siRNAs were transfected into ARPE-19 cells using lipofectamine 2000 for 12 h. Next, cells were changed mediums and incubated for another 48 h. Proteins were evaluated by Western blotting. (GenePharma, ShangHai, China).

### 4.3. Cell Viability Assay

The viability of the ARPE-19 cells were measured using cell counting kit-8 (CCK-8) assay. ARPE-19 cells were subcultured in 96-well plates at a seeding density of 2 × 10^4^ cells/well for 24 h. For assessing the effect of taurine, ARPE-19 cells were exposed to EBSS for 24 h after pretreatment in the absence or presence of varying concentrations of taurine for 1 h. Next, 100 μL CCK-8 (1:10 diluted with medium) was added into each well and cells were incubated at 37 °C for 2 h. Subsequently, the absorbance was measured at 450 nm using Multiskan GO (Thermo Fisher Scientific, Rockford, IL, USA).

### 4.4. Double Immunofluorescence Staining

Cultured cells were seeded in 24 well-plates (3 × 10^4^ cells/well) with microscope cover glasses (Citotest Labware Manufacture Co., Ltd., JiangSu, China). Cells were co-cultured with LysoTracker Green (Keygen Biotech, JiangSu, China) at a final concentration of 1 μM for 1 h. Cells were fixed with 4% paraformaldehyde (PFA) in PBS. Primary antibody staining was performed for LC3. Next, cells were washed and incubated with secondary antibodies conjugated to Alexa Fluor 594. Nuclei were stained with DAPI. All the cellular images were obtained using a confocal microscope (Olympus, Tokyo, Japan). Images were blinded and analyzed by Image-Pro Plus 6.0.

### 4.5. Annexin V/PI Double Staining

The number of dead cells was determined by FITC-AnnexinV (AV)/propidium iodide (PI) double staining (absin, ShangHai, China ABS50001A). AV (5 μL) was added to cells and incubated in the dark for 15 min. Next, the cells were washed and incubated with 1 μg/mL PI solution. Samples were then analyzed on a FACSCalibur flow cytometer (Becton, Dickinson and Company San Jose, CA, USA).

### 4.6. Western Blotting

ARPE-19 cells were lysed in a RIPA buffer (Beyotime Biotech, ShangHai, China). Protein concentrations were measured using a BCA protein assay kit (Beyotime Biotech). Equal amounts of 20 μg protein/sample were separated using 10% or 12% SDS-PAGE and the gel was transferred onto 0.45 μm PVDF membranes (Millipore, Billerica, MA, USA). The membranes were blocked in 5% BSA for 1 h at room temperature. The membranes were incubated overnight at 4 °C with primary antibodies, including calpain-1 (1:1000, Cambridge, MA, USA ab108400), calpain-2 (1:1000, ab126600), LC3 (1:1000, ab63817), Beclin-1 (1:1000, CST, Technology, Boston, MA, USA 3495), p62 (1:1000, ab109012), GRP78 (1:500, sc-376768), eIF2α (1:500, CST, 3398), Chop (1:500, CST, 5554), caspase-12 (1:500, CST, 2202), and β-actin (1:500, Proteintech, Chicago, IL, USA 66009). After washing with PBST (phosphate buffer solution and Tween-20), membranes were incubated with a secondary antibody (1:4000, Proteintech) for 1 h at room temperature. Finally, protein bands were detected using ECL reagent. The intensity of the protein band was semi-quantitatively measured with image analysis software (Image J, NIH, Betchesda, MD, USA).

### 4.7. Statistics

Quantitative data from the experiments were expressed as mean ± standard error of the mean (SEM). Statistical analysis was carried out using SPSS 21.0 software for Windows (SPSS Inc., Chicago, IL, USA). Statistical significance was determined by one-way analysis of variance (ANOVA) and a Dunnett’s post-test. In all cases, differences were considered significant if *p* < 0.05.

## 5. Conclusions

Our study suggests that ER stress, autophagy, and apoptosis could be sequentially induced by EBSS. Inhibition of ER stress and autophagy alleviated cell apoptosis. Moreover, autophagy could regulate apoptosis but not ER stress, and that autophagy and apoptosis could be regulated by ER stress. Meanwhile, calcium homeostasis was eliminated during this process. We provide evidence that calpains are the important upstream regulators to autophagy, ER stress and apoptosis. Taurine suppressed both the changes in calpain levels and the activation of ER stress, autophagy and apoptosis. Therefore, taurine inhibits starvation-triggered endoplasmic reticulum stress, autophagy, and apoptosis in ARPE-19 cells by modulating the expression of calpain-1 and calpain-2. These results further expand our understanding of the mechanism underlying EBSS and the relationship between calpains, ER stress, autophagy, and apoptosis. In future studies, it will be important to ascertain how to block autophagy and ER stress by calpain-1 and calpain-2, and also to determine taurine’s therapeutic potential to treat multiple neurodegenerative diseases associated with cell death.
